# Mammary hibernoma: A rare case report of a benign tumor

**DOI:** 10.1016/j.ijscr.2025.111061

**Published:** 2025-02-15

**Authors:** Gongzheng Li, Minggang Liu, Yuetao Lv, Ning Wang, Jian QIn, Xiuli Liu

**Affiliations:** a Jining Medical University, Jining 272067, Shandong Province, China; b Department of Breast and Thyroid Surgery, Jining First People's Hospital, Jining 272000, Shandong Province, China; c Department of Obstetrics, Jining First People's Hospital, Jining 272000, Shandong Province, China

**Keywords:** Hibernoma, Breast, Benign tumor, Brown fat, Lipoma, Case report

## Abstract

**Introduction:**

Hibernoma is a rare benign soft-tissue tumor derived from vestigial brown adipose tissue. Mammary hibernoma is a rare entity that is usually misdiagnosed clinically and radiologically as lipoma or liposarcoma.

**Presentation of case:**

We report a rare entity by dscribing the clinical, radiological and pathological features in a patient as well as the differential diagnoses and treatment. The patient underwent complete resection of the tumor and remained disease-free during 2 year follow up with no sign of any recurrence.

**Discussion:**

Hibernomais a rare benign tumor. We focus on discussing its differences from other benign or malignant tumors.

**Conclusion:**

The clinical significance of this report is to distinguish this rare tumor from other soft tissue neoplasms, both benign and malignant.

## Highlights

• A rare case of hibernoma.
• Clinical, radiological, and pathological characteristics, as well as differential diagnosis and treatment.
• The patient has a good prognosis.

## 1 Introduction

A hibernoma is a benign tumor derived from brown adipose tissue. The tumor is usually an asymptomatic and slow-growing benign mass, commonly located in various sites, such as the thigh (the most common anatomical site), back, shoulder, arm, wrist, neck, chest, arm and abdominal cavity or retroperitoneum [[1], [2], [3], [4]]. However, cases of mammary hibernoma remain extraordinarily rare; to our knowledge, fewer than 10such cases have been reported in literature written in English [1,[5], [6], [7]]. We report a rare entity by describing the clinical, radiological and pathological features in a patient as well as the differential diagnoses and treatment. All work has been reported in line with the SCARE criteria [8].

## 2 Case presentation

**Fig. 1:**
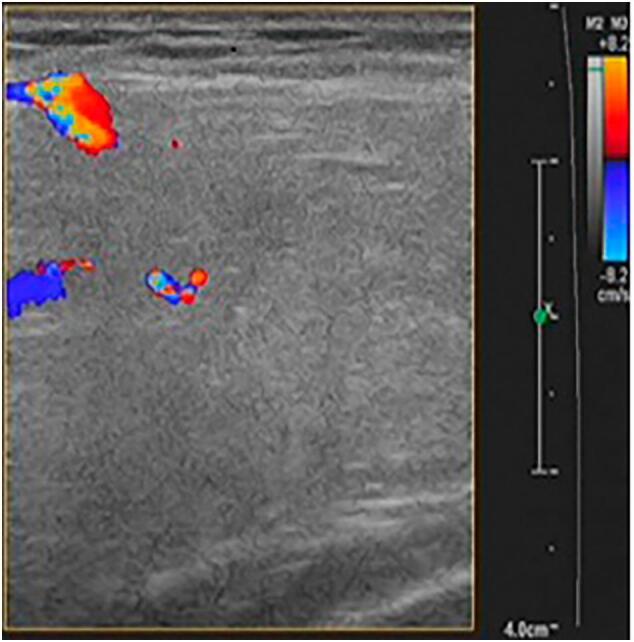
Ultrasonography revealed a hyperechoic area, with a maximum diameter of6 mm, on the upperouter quadrant of the right breast.

**Fig. 2:**
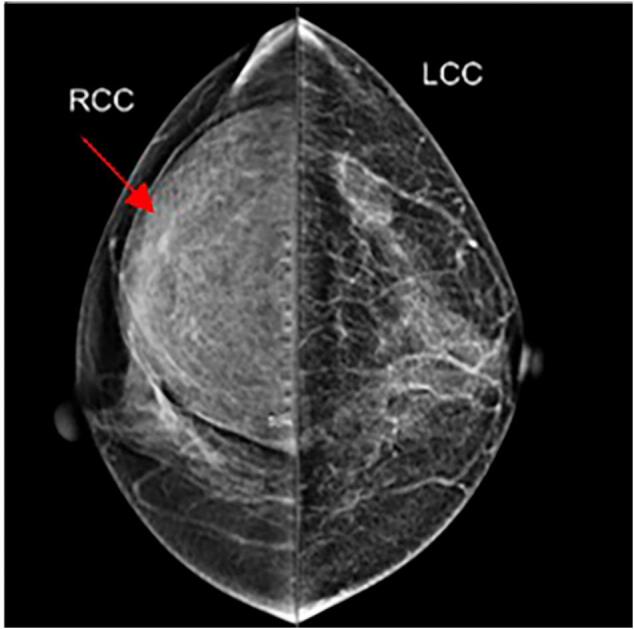
The mammogram showed a round, sharply demarcated, noncalcified lump (red arrow) in the breast. (For interpretation of the references to colour in this figure legend, the reader is referred to the web version of this article.)

**Fig. 3:**
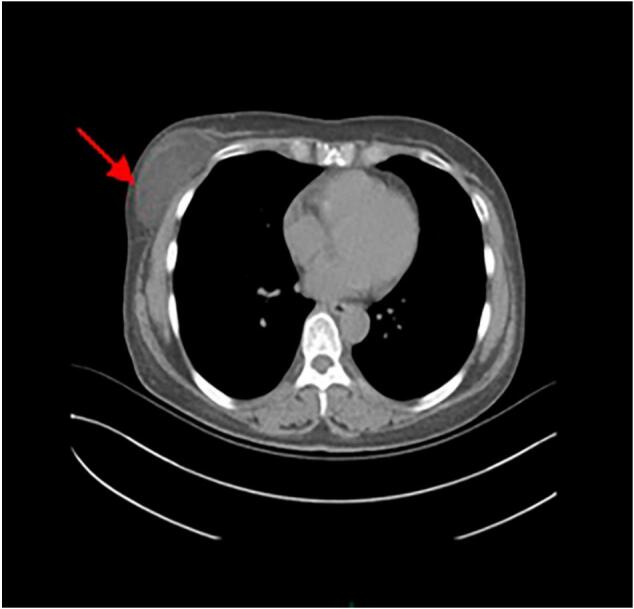
Computed tomography (CT) of the chest showed a heterogeneous low-density shadow (red arrow). (For interpretation of the references to colour in this figure legend, the reader is referred to the web version of this article.)

A 53-year-old woman was admitted to our department with a 5-year history of an asymptomatic, slow-growing, soft mass in her breast. Physical examination revealed a palpable, nontender, solitary, soft mass, approximately 10 cm × 10 cm × 8 cm in size, located on the upper-outer quadrant of the right breast. There were no other abnormalities of either breast or axilla. The laboratory examination findings, including routine blood test results, electrolyte measurements, and parameters representing coagulation, liver, renal and cardiopulmonary functions, were normal. The patient did not have a family history of breast cancer. An ultrasound examination revealed a well-circumscribed, hyperechoic noduleof dense fat on the upper outer right breast (Fig. 1). Mammography displayed a round, sharply demarcated, noncalcified lump that occupied most of the upper outer and upper inner quadrants of theright breast (Fig. 2). Computed tomography (CT) scans of the chest showed a heterogeneous low-density shadow with a maximum cross-sectional area of approximately 70 mm by 40 mm. The CT attenuation value ranged from −38 to 12 Hounsfield units (HU) (Fig. 3).

**Fig. 4:**
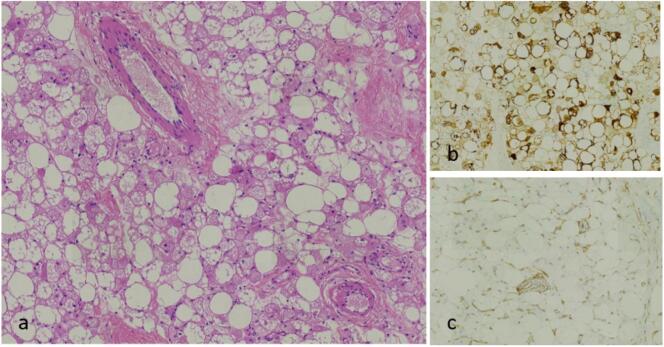
High-power microscopic image (100×, hematoxylin and eosin stain) demonstrating largepolygonal cells with abundant multivacuolated, eosinophilic cytoplasm (a). Microscopic image demonstrating positive immunohistochemical staining for S100 (b) and negative immunohistochemical staining for CD34 (c).

Microscopic, pathological examination showed abundant multivacuolated fat cells with eosinophilic cytoplasm, interspersed with regular white adipocytes and small blood vessels. In addition, immunohistochemistry was performed to confirm the diagnosis of mammary hibernoma, which was indicated by S100 protein-positive and CD34-negative staining. The histologic features were in accordance with those of ahibernoma (Fig. 4).

The patient underwent complete resection of the tumorand remained disease-free during 2 year followup with no sign of any recurrence.

## 3 Discussion

Hibernoma is a rare benign soft-tissue tumor derived from vestigial brown adipose tissue, which is rich in glycogen, phospholipids and cholesterol and believed to play a role in thermoregulation and endocrine functions [1,9]. The term “hibernoma” was first described by Grey due to its resemblance to the brown fat of hibernating animals [10,11]. There were few reports on hibernomas until the largest series of 170 cases was collected by a group from the Armed Forces Institute of Pathology (AFIP) that described the detailed clinicopathologic features [1].

A hibernoma is characterized clinically as a slow-growing, painless lump (as in our case). Large tumors may be symptomatic due to compression of adjacent structures or from abnormal weight loss associated with excessive metabolic tumor activity in the tumor; however, a few cases may even be diagnosed from accidental findings during other evaluations [1,6,12].

According to a clinicopathological study of 170 patients with hibernomas carried out byFurlong et al. in 2001, hibernomas have amean tumor size of 9.3 cm (range, 1–24 cm) and are most often diagnosed in adults, with an average age of 38 years (agerange, 2–72 years), and the morbidity is slightly higher in menthan in women [1]. Most hibernomas are subcutaneous, and only approximately 10 % areintramuscular [13].

As mentioned above, hibernomas have been reported in a wide variety of anatomical sites; when the tumors occurin the mammary region, the axilla or the axillary tail of the breast is a common site [14]. However, only a few case reports and small series have previously described this type of rare, somewhat peculiar, benign mammary tumor [[5], [6], [7],^14^,^15^]. Hibernomas usually display their own unique characteristics on medical imaging examinations. On breast ultrasonography, hibernoma typically presents as a well-defined, uniformly echogenic mass [9,15]. The lesion appears on mammography as a round, partially circumscribed, partially indistinct, noncalcified mass [6]. Thoracic computed tomography typically reveals a heterogeneous, low-density shadow that is more dense than the surrounding subcutaneous adipose tissue. On the periphery, the tumor shows a density equivalent to muscle, of 15 Hounsfield units (HU), while a hyperdense area (−3 HU) is visible in the center of the tumor [9]. On MRI, the signal strength of this lesion is between that of skeletal muscle and subcutaneous fat, and the lesion usually shows a signal strength similar to that of subcutaneous fat and a T1 signal lower than the T2 signal [14,16].

Little is known about nuclear medicine for hibernoma. Research regarding positron emission topography-computed tomography (PET-CT) has demonstrated that the anatomical location of brown adipose tissue (BAT) in adults is an active method of thermoregulation [1], which provides a better understanding of the actual embryological origins of breast hibernomas [3]. However, diagnostic biopsy is recommended for the differential diagnosis because there are no specific imaging features that allow hibernomas to be distinguished from malignant or benign soft-tissue tumors.

There are four histological classifications of hibernomas based on the properties of the matrix and the appearance of multivacuolated cells: typical, myxoid, lipoma-like, and spindle-shaped cells [1,17]. The most frequent immunohistochemical findings in the biopsy of the tumors were positive staining for S-100 and negative staining for CD34, as described in the few cases of hibernomas reported to date [1,15,18]. Cytogenetic abnormalities, including rearrangements of 11q13 and 11q21, have been described in hibernoma [1].

The differential diagnosis includes benign soft-tissue neoplasms (such as atypical lipomas, hemangiomas, and angiolipomas) and malignant, aggressive tumors (namely, well-differentiated liposarcomas, myxoid liposarcomas, and rhabdomyosarcomas) [1].

As hibernoma is considered a benign neoplasm with no risk of recurrence or malignant potential, complete surgical resection has become the mainstay of treatment, similar to that of other benign masses of the breast. [3,19,20].

## 4 Conclusion

Mammary hibernoma is a rare entity that is usually misdiagnosed clinically and radiologically as lipoma orliposarcoma. The clinical significance of this report is to distinguish this rare tumor from other soft tissue neoplasms, both benign and malignant.

## Cerdit authorship contribution statement

**Gongzheng Li:** Writing - original draft, Writing - review & editing. **Minggang Liu:** Conceptualization, Investigation. **Ning Wang:** Writing - review. **Jian QIn:** Conceptualization. **Yuetao Lv:** writing discussion and conclusion part. **Xiuli Liu:** Conceptualization, Writing – review & editing.

## Consent for publication

Written informed consent was obtained from the patient for publication of this case report and accompanying images.

## Ethical approval

This study has been approved by the Medical Ethics Committee of Jining First People's Hospital. [NO.2021伦理研第(019)号]

## Guarantor

The Guarantor is Xiuli Liu.

## Research registration number

Not applicable.

## Funding

This project is funded by the Key R&D Program of Jining (2022YXNS134).

## Declaration of competing interest

The authors declare that they have no competing interests relevant to the content of this article.
